# Individual and community-level factors associated with modern contraceptive use among adolescent girls and young women in Mali: a mixed effects multilevel analysis of the 2018 Mali demographic and health survey

**DOI:** 10.1186/s40834-020-00132-7

**Published:** 2020-10-09

**Authors:** Bright Opoku Ahinkorah, Abdul-Aziz Seidu, Francis Appiah, Eugene Budu, Collins Adu, Yaa Boahemaa Gyasi Aderoju, Faustina Adoboi, Anthony Idowu Ajayi

**Affiliations:** 1 School of Public Health, Faculty of Health, University of Technology Sydney, Ultimo, Australia; 2 Department of Population and Health, University of Cape Coast, Cape Coast, Ghana; 3 College of Public Health, Medical and Veterinary Sciences, James Cook University, Townsville, Queensland Australia; 4 Department of Health Promotion and Disability Studies, Kwame Nkrumah University of Science and Technology, Kumasi, Ghana; 5 Department of Adult Health Nursing, School of Nursing and Midwifery, University of Cape Coast, Cape Coast, Ghana; 6 Cape Coast Nursing and Midwifery Training College, Cape Coast, Ghana; 7 Population Dynamics and Sexual and Reproductive Health and Rights Unit, African Population and Health Research Center, Manga Close, Off Kirawa Road, Kitisuru, Nairobi, 00100 Kenya

**Keywords:** Modern contraceptive, Adolescent girls, Young women, Mali, DHS

## Abstract

**Background:**

Unintended pregnancy constitutes a significant public health challenge in sub-Saharan Africa and particularly among young people, who are more likely to closely space births and experience adverse obstetric outcomes. Studies on modern contraceptive use have mostly focused on women of reproductive age in general with limited attention to factors associated with modern contraceptive use among adolescents and young women (aged 15–24) in Mali. We examined the individual and community-level factors associated with modern contraceptive use among this age cohort using the 2018 Mali demographic and health survey data.

**Methods:**

We analyzed data from 2639 adolescent girls and young women, and our outcome of interest was current use of modern contraceptives. We performed descriptive analysis using frequencies and percentages and inferential analysis using mixed-effects multilevel logistic regression. The results of the mixed-effects multilevel logistic regression were presented as adjusted odds ratios with their corresponding 95% confidence intervals.

**Results:**

The prevalence of modern contraceptive use among adolescent girls and young women in Mali was 17.1% [95% CI, 15–19%]. Adolescent girls and young women who were married [aOR = 0.20, CI = 0.09–0.41], had no formal education [aOR = 0.43, CI = 0.32–0.59], in the poorest wealth quintile [aOR = 0.38, CI = 0.19–0.79] and had no children [aOR = 0.38, CI = 0.27–0.53] were less likely to use modern contraceptives. Similarly, those who had low knowledge of modern contraception [aOR = 0.60, CI = 0.42–0.85] and whose ideal number of children was six or more [aOR = 0.66, CI = 0.43–0.99] were less likely to use modern contraceptives. However, those with four or more births were more likely to use modern contraceptives [aOR = 1.85, CI = 1.24–2.77].

**Conclusion:**

Modern contraceptive use among adolescent girls and young women in Mali has improved slightly relative to the prevalence of 2012, though the prevalence is still low, compared to the prevalence in other sub-Saharan African countries and the prevalence globally. Individual-level factors such as marital status, educational level, wealth quintile, parity, ethnicity and ideal number of children were associated with the use of modern contraceptive among adolescent girls and young women in Mali. Community knowledge of modern contraceptives was found as a community-level factor associated with modern contraceptive use among adolescent girls and young women. Therefore, Mali’s Ministry of Health and Public Hygiene's Health Promotion and Education unit should prioritise and intensify contraceptive education to increase coverage of modern contraceptive use and address disparities in the use of modern contraceptives. Such education should be done, taking into consideration factors at the individual and community-level of the target population.

## Background

Demographic change is one of the major challenges facing low- and middle-income countries [1]. In low-and middle-income countries, about 16 million adolescent girls and young women (AGYW) aged 15–19 give birth annually [2, 3]. Globally, about 21 million AGYW aged 15–19 become pregnant every year [2–4]. The use of modern contraceptives, although slowly increasing, continues to be unacceptably low in sub-Saharan Africa (SSA) despite advanced awareness and knowledge among AGYW [5]. The growing use of modern contraceptives has resulted in improvements in health-related outcomes such as maternal mortality and infant mortality and improvements in education and economic outcomes, especially for AGYW [6]. In low-and middle-income countries, the prevalence of modern contraceptive use among adolescent girls and young adults is 31.6 and 43.5%, respectively [7].

The low uptake of modern contraceptives contributes considerably to the high rates of adolescent pregnancies, unsafe abortion, maternal mortality, and sexually transmitted infections [2]. The low use of contraceptives among SSA AGYW reflects, among other things, a lack of access to quality and youth-friendly services and accurate modern contraceptives information [8]. The hostile cultural context to implementing a comprehensive sexuality education known to be effective in reducing early unintended pregnancies, unsafe abortion, maternal mortality, and sexually transmitted infections and highly recommended by UNFPA [9], means more SSA AGYW will continue to bear the burden of poor reproductive health outcomes.

Acknowledging the centrality of modern contraceptives use for reproductive health outcomes, the Malian government has, since 1972, introduced various policies and strategies such as the reproductive health strategic plan (2004–2008) and national reproductive health communication program (2007–2011) and established the Association Malienne pour la Protection et la Promotion de la Famille (AMPPF), to promote the use of modern contraceptives [10]. However, these policies have focused mainly on adults and mostly neglected AGYW. Despite the introduction of modern contraceptives, the level of usage in Mali is still very low. Only 16% of married women of reproductive age (15–49 years) used modern contraceptives in 2012 [11]. Among young women, only 15.3% used modern contraceptives in 2012 [12].

Understanding factors that influence the use of modern contraceptives among AGYW is needful for designing interventions, strategies, and policies to address early pregnancies, unsafe abortion, maternal mortality and sexually transmitted infections. Previous studies on modern contraceptives use focused generally on SSA [13] and some specific countries within the region such as Ghana [2, 14], Ethiopia [15], Tanzania [5] and a joint study in Burkina Faso and Mali [12]. The study by O’Regan and Thompson [13], focused on the individual-level factors that influence modern contraceptive use and did not take into account the community-level factors and how they interact with individual-level factors to predict modern contraceptive use among AGYW. Generally, studies focusing on modern contraceptive use among AGYW in Mali are limited. A multilevel approach will contribute to the understanding of both the individual and community-level factors that predict modern contraceptive use. Using a multilevel modelling approach, this present study assesses the individual and community-level factors associated with modern contraceptive use among AGYW in Mali. Findings from this study will help to formulate useful interventions and strategies in addressing the use of modern contraceptives among AGYW.

## Methods

### Data source

Data for this study were drawn from the 2018 Mali Demographic and Health Surveys (DHS). A two multi-stage stratified cluster sampling method was employed to select the eligible respondents from rural and urban areas. Data were collected from women, men, couples and children by using different questionnaires. The details of the methodology employed in the DHS are documented in the final report of the 2018 Mali DHS [11]. The survey was designed to collect and provide data on various demographic indicators, including contraceptive use [11]. In this study, only AGYW (15–24 years) who had ever had sex and were not pregnant at the time of the study (2639) were included in our analysis. Thus, 1477 AGYW were excluded because they were either pregnant at the time of the study or had never had sex.

### Definition of variables

#### Outcome variable

The outcome variable for the study was ‘current use of modern contraceptives’. This variable was derived from a question that asked women of the type of contraceptives they were using at the time of the survey. Responses to this question were coded as “no method”, “folkloric method”, “traditional method” and “modern method”. The modern methods included female sterilization, male sterilization, intrauterine device (IUD), injectables, and implants (Norplant). The modern methods also included contraceptive pill, condoms, emergency contraception, standard day method (SDM), vaginal methods (foam, jelly, suppository), and lactational amenorrhea method (LAM). Country-specific modern methods and respondent-mentioned other modern contraceptive methods (including cervical cap, contraceptive sponge, and others) were also regarded as modern contraceptives. Periodic abstinence (rhythm, calendar method), withdrawal (coitus interruptus) and country-specific traditional methods of proven effectiveness were considered as traditional methods. Locally described methods and spiritual methods of unproven effectiveness, such as herbs, amulets, gris-gris, etc. were the folkloric methods [16, 17]. The existing DHS variable excluded women who were pregnant and those who had never had sex. For the purpose of this study, AGYW using modern methods were coded as ‘1’ while those who were not using any methods, those using traditional methods and those using folkloric methods were recoded as ‘0’.

#### Independent variables

Sixteen independent variables made up of twelve individual-level factors, and four community-level factors were considered in this study. These variables were not determined a priori; but were selected based on their theoretical relevance and practical significance with the use of modern contraceptives [17–20].

#### Individual-level factors

The individual-level factors were age (15–19, 20–24), marital status (never married, cohabiting, married, widowed/divorced), religion (Islam, other), educational level (no education, primary, secondary/higher) and employment status (not working, working). Other individual-level factors were wealth quintile (poorest, poorer, middle, richer, richest), age at first sex (less than 20, 20–24 years), parity (zero birth, one birth, two births, three or more births), and ethnicity (Bambara, Malinka, Peulh, Sarakole, Sonreal, Dogon, Touaeg, Sonoufo, Bobo, others). In addition, exposure to mass media (newspaper/magazine, radio and television), desire for more children (have another, no more, undecided) and ideal number of children (0–3, 4–5 and 6+) were also chosen as individual-level factors. Exposure to mass media was coded as ‘yes’ for AGYW who either read newspapers, listened to radio, or watched television at least once a week and less than once a week and ‘no’ for those who did not read newspaper/magazine, listen to radio or watch television at all.

#### Community-level factors

The community-level factors were residence (rural and urban), community literacy level (proportion of women who can read and write), and community socio-economic status (proportion of women in the richest household quintile). Moreover, community knowledge level of modern contraceptives (proportion of women with knowledge on modern contraceptives) was also considered as a community level factor. Community literacy level and community socio-economic status were coded as low, moderate and high while community knowledge level of modern contraceptives was coded as low and moderate.

### Statistical analyses

The data were analyzed with STATA version 14.2 for windows. First, percentages were used to describe the prevalence of modern contraceptive use. This was followed by the distribution of contraceptive use across the individual and community-levell factors. Chi-square test of independence [χ^2^] was used to assess the association between each of the factors and modern contraceptive use at a *p*-value of 0.05 (see Table 1). Finally, a two-level multilevel logistic regression analysis was performed to examine the association between individual and community-level factors and modern contraceptive use. Only variables that showed statistically significant associations with modern contraceptive use in the chi-square test were considered for the multilevel logistic regression analysis. The two-level modelling in this study implies that women were nested within clusters. Clusters were considered as random effects to cater for the unexplained variability at the community level [21].

**Table 1:** Distribution of modern contraceptive use across individual andcommunity-level factors among AGYW in Mali (Weighted)

Variables	Frequency	Percentage	Modern contraceptive use	X^**2**^ (***p***-value)
**Age**				9.4 (*p* < 0.05)
15–19	1077	40.8	13.3	
20–24	1562	59.2	19.7	
**Marital status**				67.8 (*p* < 0.001)
Never married	456	17.3	26.4	
Married	2090	79.2	14.5	
Cohabiting	48	1.8	33.2	
Widowed/divorced	45	1.7	27.8	
**Religion**				1.7 (*p* = 0.419)
Islam	2439	92.5	17.2	
Other	200	7.5	15.4	
**Educational level**				142.3 (*p* < 0.001)
No education	1394	52.8	11.9	
Primary	450	17.1	11.5	
Secondary/higher	794	30.1	29.4	
**Employment status**				7.4 (*p* < 0.05)
Not working	1300	49.3	16.8	
Working	1339	50.7	17.3	
**Wealth quintile**				90.2 (*p* < 0.001)
Poorest	361	13.7	10.1	
Poorer	487	18.5	9.5	
Middle	559	21.2	14.1	
Richer	582	22.1	21.9	
Richest	650	24.6	25.0	
**Age at first sex**				0.3 (*p* = 0.612)
Less than 20 years	2514	95.3	17.2	
20–24 years	125	4.7	15.2	
**Parity**				9.9 (*p* < 0.05)
No birth	766	29.0	12.5	
One birth	924	35.0	19.6	
Two births	603	22.9	19.1	
Three or more births	346	13.1	17.0	
**Ethnicity**				56.2 (*p* < 0.001)
Bambara	899	34.1	19.9	
Malinka	268	10.1	15.2	
Peulh	368	13.9	11.2	
Sarakole	231	8.8	13.6	
Sonreal	136	5.2	9.2	
Dogon	186	7.0	20.9	
Touaeg	55	2.1	5.0	
Sonoufo	276	10.5	23.5	
Bobo	48	1.8	14.6	
Others	172	6.5	19.6	
**Exposure to mass media**				18.6 (*p* < 0.001)
No	462	17.5	11.9	
Yes	2177	82.5	18.2	
**Desire for more children**				5.4 (*p* < 0.05)
Have another	2561	97.1	17.2	
Undecided	42	1.6	11.7	
No more	35	1.3	16.1	
**Ideal number of children**				19.2 (*p* < 0.001)
0–3	227	8.6	22.1	
4–5	1188	45.0	20.8	
6+	1223	46.4	12.6	
**Place of residence**				21.0 (*p* < 0.001)
Urban	710	26.9	25.0	
Rural	1929	73.1	14.2	
**Community literacy level**				82.3 (*p* < 0.001)
Low	924	35.0	10.1	
Moderate	892	33.8	16.9	
High	824	31.3	25.1	
**Community socio-economic status**				62.6 (p < 0.001)
Low	1595	60.4	12.6	
Moderate	190	7.2	18.3	
High	854	32.4	25.1	
**Community knowledge of modern method**				61.3 (*p* < 0.001)
Low	701	26.6	9.3	
Moderate	1938	73.3	19.9	

Source: 2018 Mali Demographic and Health Survey

Four models, consisting of the empty model (Model 0), Model 1, Model 2, and Model 3 were fitted. Model 0 showed the variance in modern contraceptive use attributed to the distribution of the primary sampling units (PSUs) in the absence of the explanatory variables. Model 1 had the individual-level factors while Model 2 contained the community-level factors. The final model (Model 3) was the complete model that had the individual and community-level factors. The STATA command ‘melogit’ was used in fitting these models. Model comparison was done using the log-likelihood ratio (LLR) and Akaike’s Information Criterion (AIC) tests. Adjusted odds ratios and associated 95% confidence intervals (CIs) were presented for all the models apart from model 0 (see Table 2). To check for high correlation among the independent variables, a test for multicollinearity was carried out using the variance inflation factor (VIF) and the results showed no evidence of high collinearity (Mean VIF = 1.69, Maximum VIF = 3.84, and Minimum VIF = 1.06). Sample weight (v005/1,000,000) and SVY command were used to correct for over and under-sampling and the complex survey design and generalizability of the findings, respectively.

**Table 2:** Mixed effects results on individual and community-level factors associated with modern contraceptive use among AGYW in Mali

Variables	Model 0	Model 1 aOR[95%CI]	Model 2 aOR[95%CI]	Model 3 aOR[95%CI]
**Age**				
15–19		0.90 [0.68–1.19]		0.90 [0.68–1.20]
20–24		1		1
**Marital status**				
Not married		0.58 [0.28–1.22]		1.59 [0.28–1.24]
Married		0.19^***^[0.09–0.40]		0.20^***^[0.09–0.41]
Cohabiting		1		1
Widowed		0.60 [0.24–1.54]		0.61 [0.24–1.56]
**Educational level**				
No education		0.41^***^[0.31–0.55]		0.43^***^[0.32–0.59]
Primary		0.43^***^[0.29–0.62]		0.43^***^[0.29–0.63]
Secondary/higher		1		1
**Employment status**				
Not working		0.95 [0.74–1.22]		0.97 [0.75–1.24]
Working		1		1
**Wealth quintile**				
Poorest		0.37^***^[0.21–0.65]		0.38^**^[0.19–0.79]
Poorer		0.46^***^[0.28–0.75]		0.45^*^[0.23–0.86]
Middle		0.80 [0.53–1.19]		0.77 [0.43–1.36]
Richer		1.15 [0.83–1.59]		1.11 [0.74–1.67]
Richest		1		1
**Parity**				
Zero birth		0.37^***^[0.26–0.52]		0.38^***^[0.27–0.53]
One birth		1		1
Two births		1.38 [1.00–1.92]		1.40^*^ [1.01–1.94]
Three births		1.84^**^[1.23–2.75]		1.85^**^[1.24–2.77]
Four or more births				
**Ethnicity**				
Bambara		0.86 [0.56–1.31]		0.86 [0.55–1.32]
Malinka		0.75 [0.43–1.31]		0.77 [0.44–1.34]
Peulh		0.52^*^[0.30–0.89]		0.53^*^[0.31–0.91]
Sarakole		0.66 [0.36–1.21]		0.68 [0.37–1.24]
Sonreal		0.39^**^[0.21–0.70]		0.43^**^[0.23–0.77]
Dogon		1.35 [0.75–2.46]		1.34 [0.74–2.43]
Touaeg		0.34^**^[0.16–0.73]		0.42^*^[0.19–0.91]
Sonoufo		1		1
Bobo		0.83 [0.30–2.28]		0.80 [0.29–2.20]
Others		0.83 [0.45–1.52]		0.83 [0.45–1.51]
**Exposure to mass media**				
No		0.92 [0.63–1.33]		0.96 [0.66–1.39]
Yes		1		1
**Desire for more children**				
Have another		1		1
Undecided		0.35^*^ [0.13–0.98]		0.36 [0.13–1.00]
No more		0.93 [0.31–1.55]		0.98 [0.43–2.21]
**Ideal number of children**				
0–3		1		1
4–5		0.97 [0.65–1.42]		0.93 [0.63–1.38]
6+		0.67 [0.44–1.01]		0.66^*^[0.43–0.99]
**Place of Residence**				
Urban			1	1
Rural			1.28 [0.83–1.99]	1.15 [0.72–1.83]
**Community literacy level**				
Low			0.39^***^[0.25–0.63]	0.77 [0.45–1.27]
Moderate			0.59^***^[0.40–0.86]	0.79 [0.54–1.16]
High			1	1
**Community socio-economic status**				
Low			0.67 [0.41–1.10]	1.18 [0.67–2.08]
Moderate			0.84 [0.46–1.54]	1.13 [0.61–2.11]
High			1	1
**Community knowledge of modern method**				
Low			0.43^***^[0.30–0.60]	0.60^**^ [0.42–0.85]
Moderate			1	1
**Random effect result**				
PSU variance (95% CI)	0.83 (0.54–1.29)	0.31 (0.15–0.67)	0.42 (0.22–0.78)	0.29 (0.13–0.64)
ICC	20.2%	8.7%	11.2%	8.1%
LR Test	χ^2^ = 65.73, *p* < 0.001	χ^2^ = 12.15, *p* < 0.05	χ^2^ = 21.12, *p* < 0.001	χ^2^ = 10.73, *p* < 0.05
Wald chi-square	Reference	206.79^***^	76.53^***^	209.73^***^
Model fitness				
Log-likelihood	− 1096.70	−972.84	− 1056.96	−967.29
AIC	2197.41	2005.68	2129.91	2005.58
BIC	2209.16	2182.02	2176.94	2218.19
N	2639	2639	2639	2639

Source: 2018 Mali Demographic and Health Survey

^*^p < 0.05, ^**^p < 0.01, ^***^p < 0.001

*1* Reference category, *PSU* Primary Sampling Unit, *ICC* Intra-Class Correlation, *LR Test* Likelihood ratio Test, *AIC* Akaike’s Information Criterion; Bayesian information criterion

### Ethical approval

This was a secondary analysis of data, and therefore no further approval was required for this study since the data is secondary and is available in the public domain. However, the source of data (DHS) reports that ethical clearance was obtained from the Ethics Committee of ORC Macro Inc. as well as Ethics Boards and the Ministry of Health of Mali. The DHS follows the standards for ensuring the protection of respondents’ privacy. Inner City Fund (ICF) International ensured that the survey complies with the U.S. Department of Health and Human Services regulations for the respect of human subjects. To have access to the data, the authors officially requested and obtained access from MEASURE DHS to download and use the dataset.

## Results

### Distribution of modern contraceptive use across individual and community-level factors among AGYW in Mali

Table 1 presents the results of the distribution of modern contraceptive use across the individual and community-level factors of AGYW in Mali. Almost 59% (59.2%) of the respondents were aged 20–24. The majority of respondents were married (79.2%), Muslims (92.5%), exposed to media (82.5%), desired for more children (97%), and had their first sex before the age of 20 (95.3%). Over half of them had no formal education (52.8%), and were working (50.7%). Only 24.6% were in the richest wealth quintile. The results further showed that 35% of the AGYW had parity one, while 13.1% had three or more births. Also, our analysis shows that 34.1% were of the Bambara ethnic group. About 46% of them considered 6+ as their ideal number of children. Seventy-three out of every 100 AGYW resided in rural areas, 35% were in the low community literacy level category, 60% were in the low-socio-economic status, and 73.3% had moderate community-level knowledge on modern contraception. The chi-square test results also revealed that apart from age at first sex and religious affiliation, all the independent variables had statistically significant associations with modern contraceptive usage.

### Prevalence of modern contraceptive use among AGYW in Mali

**Fig. 1:**
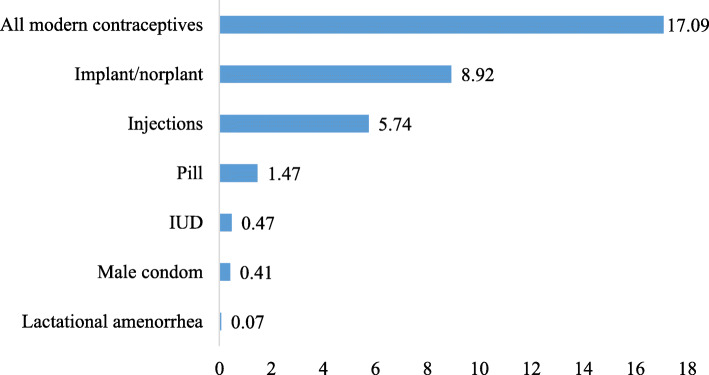
Prevalence of Modern contraceptive use among adolescent girls and young women in Mali

Figure 1 displays the results on the prevalence of modern contraceptive use among AGYW in Mali. Our analysis shows that 17.1% [95% CI, 15–19%] of AGYW were using modern contraception, 8.9% were using Implant/Norplant.

### Measures of association (fixed effects)

The study found a statistically significant association between individual and community-level factors and modern contraceptive use. Specifically, married AGYW had lower odds of using modern contraception [aOR = 0.20, CI = 0.09–0.41], compared with those who were cohabiting. AGYW with no formal education [aOR = 0.43, CI = 0.32–0.59] and primary education [aOR = 0.43, CI = 0.29–0.63] were less likely to use modern contraceptives compared to those with secondary/higher education. Those in the poorest [aOR = 0.38, CI = 0.19–0.79] and poorer wealth quintile [aOR = 0.45, CI = 0.23–0.86] were less likely to use modern contraceptives compared to those in the richest wealth quintile. AGYW with parity zero were less likely to use modern contraceptives [aOR = 0.38, CI = 0.27–0.53] while those with four or more births were more likely to use contraceptives [aOR = 1.85, CI = 1.24–2.77], compared to those with parity one. AGYW whose ideal number of children was six or more children had lower odds of using modern contraceptives [aOR = 0.66, CI = 0.43–0.99], compared with those who desired up to three children. Modern contraceptive use was low among women who belonged to the Peulh, Sonreal and Touaeg ethnic groups. A lower odd of modern contraceptive use was found among AGYW who had low knowledge on modern contraception [aOR = 0.60, CI = 0.42–0.85] (see Table 2).

### Measures of variation (random effects)

In model 0, the clustering of the primary sampling units (PSUs) accounted for substantial variations in the odds of modern contraceptive use (σ2 = 0.83, 95% CI 0.54–1.29). Model 0 showed that 20.2% of the total variation in modern contraceptive use was attributed to the variance between clusters (ICC = 0.22). The between-cluster variance showed a decrease from 20.2 to 8.7% from Model 0 to Model 1 (individual level only model). From Model I, the ICC increased to 11.2% in Model 2 (community level only model), but decreased to 8.1% in the complete model (Model III), where all the independent variables were considered. This indicates that differences in the clustering of the PSUs account for the variations in modern contraceptive use. The highest log-likelihood (− 967.29) and the lowest AIC (2005.58) were used to determine the best fit model (see Table 2).

## Discussion

This study assessed the individual and community-level factors associated with modern contraceptive use among AGYW in Mali. The prevalence of modern contraceptive use was low, with only 17.1% of AGYW using any modern methods at the time of the survey. Marital status, educational level, wealth quintile, parity, ethnicity and ideal number of children were identified as individual-level factors associated with modern contraceptive use among AGYW in Mali. While only community knowledge level of modern contraceptives had a significant association with modern contraceptive use, of all the community-level factors.

Findings on the prevalence of modern contraceptive use is in line with the findings obtained in a previous study carried out to examine possible indicators of modern contraceptive use among young women’s modern contraceptive use in Burkina Faso and Mali. The study found that in Mali, modern contraceptive use increased from 9.4% in 2001 to 10.2% in 2006 to 15.3% in 2012 [12]. The prevalence in our study also indicates an improvement from the prevalence of 15.3% in 2012, and an indication that the Government of Mali’s objective of ensuring an increase in the rate of contraceptive use in Mali, from 9.9% in 2012 to at least 15% by 2018, through the reduction of unmet need for family planning and by targeting teens and young adults (ages 15 to 24) [22] has been met. However, it must be added that this target was for all women in Mali and not AGYW only. It is therefore important that more effort is put in place to enhance the use of modern contraceptives among AGYW despite improvement from 2012.

We found that AGYW who were married had lower odds of using modern contraception compared with those who were cohabiting. This result is in contrast to earlier findings that noted that, being married remained significantly associated with ever use of a modern contraceptive method, with married women being more than twice as likely to have ever used modern contraception, compared with unmarried women in Ghana [23]. However, this study was conducted among all women of reproductive age (15–49), compared to those aged 15–24 used in the current study. It has been argued that married women, especially those at youth stage and adolescents, are confronted with pressure to have a child soon after marriage, which exposes them to pregnancy even if they had wished to delay pregnancy [3, 24]. Also, it is well known that cultural values and gender norms strongly influence fertility desires and family planning needs, and in many settings, women are expected to give birth to at least one child before adopting contraception [25–27]. As such, it is not surprising that AGYW who were married were less inclined to modern contraceptive usage in the present study.

Additionally, the present study also revealed that AGYW with no formal education and primary education were less likely to use modern contraceptives compared to those with secondary/higher education. Evidence from Tanzania also indicated that unmarried-sexually active women who had reached the university level of education were three times more likely to use modern contraception compared with those with no education [5]. It has been argued that advancement in education has a potential role in shaping individuals’ perceptions and knowledge about modern contraceptives, which aid them in overcoming misconceptions against modern contraception use [28]. Therefore the youth who were less educated had a lower chance of using modern contraceptives probably due to misconceptions [8].

Consistent with previous studies [28, 29], the study also revealed that those with poorest and poorer wealth quintiles were less likely to use modern contraceptives compared to those with richest wealth quintile. A study in Malawi also indicated that women in the highest wealth quintiles were more likely to use contraception as compared to those in the poorest income categories [29]. The influence of wealth status on modern contraceptives usage was also confirmed by a Ugandan-based study which noticed that women in higher wealth quintiles were significantly more likely to use postpartum family planning compared to those within the lowest wealth quintiles [30]. The poor and poorest would be less inclined to modern contraceptives usage partly because, theoretically, they might not have the perceived behavioural control to utilize modern contraceptives when they are financially not viable to enable them to buy these modern contraceptives [31].

Additionally, it was revealed that AGYW with no children were less likely to use modern contraceptives while those with four or more births were more likely to use contraceptives, compared to those with parity one. A Jordan-based study noted that as the number of children increased, women were more likely to rely on modern contraceptive methods than on traditional ones [32]. The result is also in line with a study by Withers, Kano and Pinatih, [33] which indicated that the number of living children was associated with contraceptive use and that an increase in the number of living children increased the odds of using contraception by 12%. Similarly, Bulto, Zewdie and Beyen [34] noted that women who had three, four and five or more ever born child were more likely to have demand for long-acting and permanent methods of contraception (LAPMs), when compared to those who had no child in Ethiopia. This result could be likened to a study done by Haile and Fantahun [35], which showed women with 1–3 and 4–12 children were having fifty-one and six times higher demand than those who had no child respectively in Ethiopia. A recent study in the same country also found that women who had 2–3 children were almost three times more likely to have demand for modern methods of contraception than women who had no and one child [36]. In explaining the plausible reason accounting for this observation, Kebede, Abaya, Merdassa and Bekuma [36] contended that due to the more child the woman is having, the more likely she wants to space or limit the number of children and the more she was using contraceptives.

With regard to the ideal number of children, we observed that AGYW whose ideal number of children was six or more had lower odds of using modern contraceptives compared with those who desired 0–3 children. Similarly, in Indonesia, a study found that women who wanted more children had a lower odds of using contraceptives compared to those who reported not wanting more children [33]. Within the African setting, a study has shown that some women perceive that modern contraceptive use can cause infertility by destroying their reproductive system or rendering the womb weak when used at a young age or before bearing at least one child [11, 37]. Another argument also advanced that women use modern contraceptives for spacing births, and as such, those who do not have children tend not to utilize modern contraceptives [38]. In contrast, Chandra-Mouli, McCarraher, Phillips, Williamson and Hainsworth [39] explained that women, mostly the unmarried, use modern contraceptives to prevent pregnancies. This perception held on modern contraceptives could explain why young Malian women desiring to have a higher number of children will tend not to use modern contraceptives.

The current study also noted that modern contraceptive use was low among adolescent girls and young women who belonged to the Peulh, Sonreal and Touaeg ethnic groups. Several factors to have influenced modern contraceptive usage have been observed, including knowledge, religious beliefs, financial considerations, fear of side effects, partner’s disapproval, and problems with decision-making at home [40, 41]. Furthermore, Afriyie and Tarkang [42] reported that women without problems in decision-making tend to be self-efficacious, which empowered them to be responsible for their health, hence take decisions regarding modern contraception usage. They also added that having a supportive husband in the decision-making process regarding the use of modern contraception influence its use. As such, we agree with these researchers that AGYW belonging to Peulh, Sonreal and Touaeg ethnic groups were less likely to use modern contraceptives probably because they had less support from their spouses or restricted by their religious beliefs. However, further studies to holistically assess ethnicity and modern contraceptive use among Mali AGYW will be needful since these studies failed to unravel the reasons for the disparity in modern contraceptive usage, stratified by ethnicity.

Finally, consistent with other studies [5, 43], the present study revealed that AGYW who have low knowledge of modern contraception, were less likely to use modern contraceptives as compared to those with moderate to high knowledge of modern contraception. Williamson, Parkes, Wight, Petticrew and Hart [43], in their systematic review about limits to modern contraceptive use among AGYW in developing countries, concluded that the use of hormonal methods was limited by lack of knowledge. The result is also in line with findings by Nsanya, Atchison, Bottomley, Doyle and Kapiga [5], which noted that the odds of using modern contraception increased with higher knowledge about contraception among Tanzanian women aged 15–19. A probable reason for our observation is that with less knowledge of modern contraceptives, AGYW would misconstrue the immediate and long term side effects of modern contraceptives on fertility and health implications associated with its use, which could dissuade them from its utilization [11, 40]. This finding also underscores the need to educate young people about accurate contraceptive information.

### Strengths and limitations

This study assessed the individual and community-level factors associated with the use of modern contraceptives among AGYW in Mali by adopting sound analytical procedure, specifically the mixed-effects multilevel analysis, hence making the study results robust. Additionally, our study also used dataset obtained from the 2018 DHS of Mali, hence presenting a current and accurate picture of modern contraceptive use among AGYW in the country. Additionally, the study depended on a survey of a relatively large population-based sample with national coverage. However, the study has some limitations. Firstly, due to the retrospective reporting of modern contraceptives used, there may be some recall bias in the data. Also, issues with contraception are somehow sensitive; therefore, respondents may respond to the questions with the intention of craving a positive image about themselves, hence making the data liable to social desirability bias. Again, because of the cross-sectional design of the study, the analysis can only provide evidence of statistical association, and cause-effect relationships cannot be inferred. Finally, the use of log-likelihood (L2) and AIC has its inherent weakness. With the log-likelihood (L2), the conditional test by subtracting L2 and the number of free parameters between models with T and T + 1 classes do not have an asymptotic chi-squared distribution [44]. With the use of AIC, we acknowledge the limiatation that AIC relies on an asymptotic approximation that may not hold for a given finite data set, compared to BIC which relies on the assumption that the model errors are independent and normally distributed. Notwithstanding, both AIC and BIC provide measures of model performance that account for model complexity [45].

## Conclusion

Modern contraceptive use among adolescent girls and young women in Mali has improved slightly compared to the prevalence recorded in 2012, though the prevalence is still low, compared to the prevalence in other sub-Saharan African countries and the prevalence globally. Marital status, educational level, wealth quintile, parity, ethnicity and ideal number of children were identified as individual-level factors associated with modern contraceptive use among adolescent girls and young women in Mali. Community knowledge of modern contraceptives was found as a community-levelfactor associated with modern contraceptive use amongAGYW. Therefore, Government of Mali, through its policymakers on family planning in collaboration with Mali's Ministry of Health and Public Hygiene's Health Promotion and Education unit, should intensify mass education to address disparities in modern contraceptive use among AGYW. Such education should be done, taking into consideration factors at the individual and community levels of the target population. These factors should include marital status, educational level, wealth quintile, parity, ethnicity, ideal number of children and community knowledge of modern contraceptives.

## Data Availability

The dataset supporting the conclusions of this article is available online at https://dhsprogram.com/data/

## Abbreviations

AIC: Akaike’s Information Criterion
ICF: Inner City Fund
PSU: Primary sampling units
IUD: Intrauterine contraceptive device
SDM: Standard day method
AGYW: Adolescent girls and young women
DHS: Demographic and Health Survey
ICC: Intra-Class Correlation
LR: Likelihood ratio Test
AIC: Akaike’s Information Criterion; Bayesian information criterion

## References

[1] May J, Guengant J (2014). Les défis démographiques des pays sahéliens. Ĕtvdes.

[2] Appiah F, Seidu A-A, Ahinkorah BO, Baatiema L, Ameyaw EK (2020). Trends and determinants of contraceptive use among female adolescents in Ghana: analysis of 2003–2014 demographic and health surveys. SSM - Population Health.

[3] UNFPA (2015). Girlhood, not motherhood: preventing adolescent pregnancy.

[4] Kirchengast S (2016). Teenage pregnancies: a worldwide social and medical problem. An analysis of contemporary social welfare issues.

[5] Nsanya MK, Atchison CJ, Bottomley C, Doyle AM, Kapiga SH (2019). Modern contraceptive use among sexually active women aged 15–19 years in North-Western Tanzania: results from the adolescent 360 (A360) baseline survey. BMJ Open.

[6] United Nations Population Division (2017). World family planning 2017 highlights. Department of Economic and Social Affairs.

[7] Li Z, Patton G, Sabet F, Zhou Z, Subramanian SV, Lu C (2020). Contraceptive use in adolescent girls and adult women in low-and middle-income countries. JAMA Netw Open.

[8] Ajayi AI, Nwokocha EE, Akpan W, Adeniyi OV (2016). Use of non-emergency contraceptive pills and concoctions as emergency contraception among Nigerian University students: results of a qualitative study. BMC Public Health.

[9] UNFPA (2014). Adding it up the benefits of investing in sexual and reproductive health care.

[10] Stanley YP, Guèye M, Konatè M (2011). The use of family planning methods in Mali. The how and why of taking action.

[11] Institut National de la Statistique (INSTAT) ICF (2019). 2018 Mali demographic and health survey key findings.

[12] O’Regan A, Thompson G (2017). Indicators of young women’s modern contraceptives use in Burkina Faso and Mali from demographic and health survey data. Contraception Reprod Med.

[13] Bankole A, Audam S (2011). Fertility preference and contraceptive use among couples in sub-Saharan Africa. Afr Popul Stud.

[14] Tekelab T, Melka AS, Desalegn Wirt U (2015). Predictors of modern contraceptive methods use among married women of reproductive age groups in Western Ethiopia: a community based cross-sectional study. BMC Womens Health.

[15] Amalba A, Mogre V, Appiah MN, Mumuni WA (2014). Awareness, use and associated factors of emergency contraceptive pills among women of reproductive age (15–49 years) in tamale, Ghana. BMC Womens Health.

[16] Corsi DJ, Neuman M, Finlay JE, Subramanian SV (2012). Demographic and health surveys: a profile. Int J Epidemiol.

[17] Aviisah PA, Dery S, Atsu BK, Yawson A, Alotaibi RM, Rezk HR (2018). Modern contraceptive use among women of reproductive age in Ghana: analysis of the 2003–2014 Ghana demographic and health surveys. BMC Womens Health.

[18] Debebe S, Limenih MA, Biadgo B (2017). Modern contraceptive methods utilization and associated factors among reproductive aged women in rural Dembia District, Northwest Ethiopia: community based cross-sectional study. Int J Reprod BioMed.

[19] Ejembi CL, Dahiru T, Aliyu AA (2015). Contextual factors influencing modern contraceptive use in Nigeria. ICF.

[20] Lasong J, Zhang Y, Gebremedhin SA, Opoku S, Abaidoo CS, Mkandawire T (2020). Determinants of modern contraceptive use among married women of reproductive age: a cross-sectional study in rural Zambia. BMJ Open.

[21] Solanke BL, Oyinlola FF, Oyeleye OJ, Ilesanmi BB (2019). Maternal and community factors associated with unmet contraceptive need among childbearing women in northern Nigeria. Contracept Reprod Med.

[22] FP2020 (2017). Mali FP2020 revitalized commitment 2017.

[23] Aryeetey R, Kotoh AM, Hindin MJ (2010). Knowledge, perceptions and ever use of modern contraception among women in the Ga East District, Ghana. Afr J Reprod Health.

[24] UNFPA (2012). Marrying too young end child marriage. New York, USA.

[25] Dynes M, Stephenson R, Rubardt M, Bartel D (2012). The influence of perceptions of community norms on current contraceptive use among men and women in Ethiopia and Kenya. Health Place.

[26] Adams MK, Salazar E, Lundgren R (2013). Tell them you are planning for the future: gender norms and family planning among adolescents in northern Uganda. Int J Gynaecol Obstet.

[27] Kane S, Kok M, Rial M (2016). Social norms and family planning decisions in South Sudan. BMC Public Health.

[28] Mardi A, Ebadi A, Shahbazi S (2018). Factors influencing the use of contraceptives through the lens of teenage women: a qualitative study in Iran. BMC Public Health.

[29] Adebowale SA, Adedini SA, Ibisomi LD, Palamuleni ME (2014). Differential effect of wealth quintile on modern contraceptive use and fertility: evidence from Malawian women. BMC Womens Health.

[30] Rutaremwa G, Kabagenyi A, Wandera SO, Jhamba T, Akiror E, Nviiri HL (2015). Predictors of modern contraceptive use during the postpartum period among women in Uganda: a population-based cross sectional study. BMC Public Health.

[31] Ajzen I (1991). The theory of planned behaviour. Organ Behav Hum Decis Process.

[32] Almalik M, Mosleh S, Almasarweh I (2018). Are users of modern and traditional contraceptive methods in Jordan different?. East Mediterr Health J.

[33] Withers M, Kano M, Pinatih GNI (2010). Desire for more children, contraceptive use and unmet need for family planning in a remote area of Bali, Indonesia. J Biosoc Sc.

[34] Bulto GA, Zewdie TA, Beyen TK (2014). Demand for long acting and permanent contraceptive methods and associated factors among married women of reproductive age group in Debre Markos town, north West Ethiopia. BMC Womens Health.

[35] Haile A, Fantahun M (2012). Demand for long acting and permanent contraceptive methods and associated factors among family planning service users, Batu Jira town, Central Ethiopia. Ethiop Med J.

[36] Kebede A, Abaya SG, Merdassa E, Bekuma TT (2019). Factors affecting demand for modern contraceptives among currently married reproductive age women in rural Kebeles of Nunu Kumba district, Oromia, Ethiopia. Contracept Reprod Med.

[37] Sedlander E, Bingenheimer JB, Thiongo M, Gichangi P, Rimal RN, Edberg M (2018). “They destroy the reproductive system”: exploring the belief that modern contraceptive use causes infertility. Stud Fam Plan.

[38] Achana FS, Bawah AA, Jackson EF, Welaga P, Awine T, Asuo-mante E (2015). Spatial and socio-demographic determinants of contraceptive use in the upper east region of Ghana. Reprod Health.

[39] Chandra-Mouli V, McCarraher DR, Phillips SJ, Williamson NE, Hainsworth G (2014). Contraception for adolescents in low and middle income countries: needs, barriers, and access. Reprod Health.

[40] Saluja N, Sharma S, Choudhary S, Gaur D, Pandey S (2011). Contraceptive knowledge, attitude and practice among eligible couples of rural Haryana. Internet J Health.

[41] Frini HO, Nabag WOM (2013). The knowledge and determinant factors of contraceptive use among married Sudanese women. App Sci Report.

[42] Afriyie P, Tarkang EE (2019). Factors influencing use of modern contraception among married women in ho west district, Ghana: descriptive cross-sectional study. Pan Afr Med J.

[43] Williamson LM, Parkes A, Wight D, Petticrew M, Hart GJ (2009). Limits to modern contraceptive use among young women in developing countries: a systematic review of qualitative research. BioMed Central Reprod Health.

[44] Cousineau D, Allan T (2015). Likelihood and its use in parameter estimation and model comparison. Mesure et évaluation en éducation.

[45] Kriegeskorte N (2015). Crossvalidation in brain imaging analysis. Biorxiv..

